# Tuning the Catalytic Activity of Ir@Pt Nanoparticles Through Controlling Ir Core Size on Cathode Performance for PEM Fuel Cell Application

**DOI:** 10.3389/fchem.2018.00299

**Published:** 2018-07-26

**Authors:** Hao-Bo Zheng, Lu An, Yuying Zheng, Chong Qu, Yanxiong Fang, Quanbing Liu, Dai Dang

**Affiliations:** ^1^School of Chemical Engineering and Light Industry, Guangdong University of Technology, Guangzhou, China; ^2^Department of Materials Science and Engineering College of Engineering, Peking University, Beijing, China

**Keywords:** core size effect, core-shell structure, low Pt loading, membrane electrode assembly, fuel cell

## Abstract

Pulse electrochemically synthesis of a series of core-shell structured Ir@Pt/C catalysts in cathode catalysts layer are achieved to fabricate membrane electrode assemblies (MEA) with cathode ultra-low Pt loading. The single cell performance of the MEAs in a H_2_/air PEMFC greatly rely on the sizes of the Ir core nanoparticle, and the optimum activity occurs with Ir core size of 4.1 nm. The cathode MEA with core-shell structured catalysts with optimal Ir core size exhibited excellent performance in a H_2_/air single fuel cell, comparable to that of a commercial Pt/C MEA (Johnson Matthey 40% Pt), even though the Pt loading in Ir@Pt was only 40% that of the commercial Pt cathode (0.04 vs. 0.1 mg cm^−2^). The catalysts were characterized by X-ray diffraction, X-ray photoelectron spectroscopy (XPS) and scanning transmission electron microscopy. Based on the characterization results, especially from XPS, we suggest that the effect of Ir core particle size on MEA performance may arise from the interactions between the Pt shell and the Ir core. The XPS results showed that the Ir@Pt/C-300 catalyst has the highest Pt^0^ fraction among the four tested samples. This work demonstrates the alternative to enhance the cathode performance in single cell of Pt-based core-shell structured catalysts by varying size of the core metal under the Pt shell.

## Introduction

With the ever-growing concerns of global environment and increasing consumption of fossil fuels, the development of new energy conversion and storage system is of great significance across the world (Stamenkovic et al., 2016; Dai et al., 2017; Zhang et al., 2017, 2018; Zhao et al., 2018). Proton exchange membrane fuel cells (PEMFCs), regarded as the most encouraging clean power sources for future automobile, have been receiving unprecedented attentions due to their high energy efficiency, zero emission, and remarkable environmental acceptability (Debe, 2012; Dang et al., 2018). Nonetheless, there are some major issues that still slow down the pace of commercialization of PEMFCs (Shao et al., 2016; Tian et al., 2016). For example, the high loading of platinum and high cost of the Pt catalyst with sluggish oxygen reduction kinetics at cathode (Chen et al., 2015). However, the vital factor that affects the fuel cell performance is the catalyst layer within the membrane electrode assembly (MEA), which consists of Pt/carbon black and Nafion® ionomer mixture (Kim et al., 2013). In general, the conventional MEA (prepared by catalyst coated membrane method, CCM) requires high Pt content (20–60%) to satisfy the chemical reactions needs. Whereas, a large fraction of Pt catalysts is not utilized by this approach (CCM) because Pt active nanoparticles are either losing ion contact with solid electrolyte or unable to access to the electronic path with carbon (Gasteiger et al., 2005). Accordingly, reducing the Pt loading within the electrode as well as without sacrificing on the cell performance is in harsh demand for PEMFC market, which not only requires the development of novel catalysts, but also strongly recommends a satisfactory nanostructured catalyst layer in the MEA.

Core-shell structured catalysts were introduced and developed for decades to find an answer. The core structure effect, including the shape, particle size, porosity, and composition were thoroughly investigated, and has shown significant impact on the oxygen reduction reaction (ORR) activity (Gan et al., 2012; Yang et al., 2013; Chen et al., 2014; Lu et al., 2014; Wittkopf et al., 2014; Zhang et al., 2014; Takimoto et al., 2017).

In recent years, Ir@Pt/C catalysts have been made to use in acid media for methanol oxidation and oxygen reduction reaction with the satisfactory results (Strickler et al., 2017). However, to the best of our knowledge, there is still no research about the core size effect in terms of Ir@Pt/C series catalysts tested in the single cell. Meanwhile, the Ir@Pt/C catalysts operated in a real PEM fuel cell cathode electrode environment has seldom been reported. Inspired by the continued achievements, we demonstrate a facile synthesis route for the core-shell structured catalyst within cathode catalysts layer, which is realized by pulse electrochemical deposition (PED) method. Intriguingly, we discovered that the Ir cores with different sizes may had some pronounced effects on the ORR performance. The Ir@Pt/C MEA prepared in the present work with the ultra-low Pt loading of 0.04 mg cm^−2^ at cathode is outperformed than that of the commercial JM Pt/C with Pt loading of 0.1 mg cm^−2^.

## Experimental

### Ir/C catalyst preparation

Ir/C, the carbon-supported Ir core (Ir/C; 20 wt.% of Ir loading), was prepared by an impregnation-reduction method previously reported by our group (Dang et al., 2014). Briefly, IrCl_3_ and pretreated XC-72R carbon black were both added in ethanol to form mixture. The mixture was magnetically stirred at 70°C for 8 h to eliminate ethanol. The black powder was then placed in a ceramic boat and heated in a tubular furnace under flowing H_2_ for 2 h at 180, 240, 300, 400, and 500°C, respectively. Then the heat-treated catalysts were labeled as Ir/C-180, Ir/C-240, Ir/C-300, Ir/C-400, and Ir/C-500.

### Synthesis of Ir@Pt/C MEA

The two-stage strategy was adopted to obtain cathode Ir@Pt/C electrode. Firstly, home-made Ir/C catalyst was mixed with 5 wt.% Nafion® ionomer solution (DuPont, USA) and isopropanol, then sonicated for 30 min to achieve homogeneous dispersion. The mixture was then directly sprayed onto one side of the membrane (Nafion® 212, Dupont) possessing an area of 5 cm^2^. The weight ratio of the Ir/C catalyst to dry Nafion® was 2.5:1. The Ir loading was 0.039 mg cm^−2^. Secondly, A laboratory device was used to prepare the Ir@Pt/C based MEA. The Ir/C based MEA was placed in a fixed area of 5 cm^2^, with the Ir/C serving as the working electrode, platinum wire and an Ag/AgCl electrode (3 M KCl) as the counter and reference electrode, respectively. Peak current densities were set to 30 mA cm^−2^, 0.3 ms of the time on and 0.15 ms of time off for PED process. The Pt loadings of the Ir@Pt/C cathodes were 0.04 mg cm^−2^ for each of them, which were detected by atomic absorption spectroscopy (AAS).

The JM Pt/C MEA were prepared by the catalyst coated membrane method previously reported by our group using JM Pt/C (Johnson Matthey, 40%Pt) catalyst for both anode and cathode.

### Fuel cell measurements

The MEA was assembled by putting gas diffusion layers, which were prepared by spraying a carbon-Teflon® mixture on the pretreated carbon paper, on the anode and cathode side.

The MEA was tested using a Fuel Cell Testing System (Arbin Instruments, USA). The cell temperature was maintained at 70°C. H_2_ and air, as the fuel and oxidant gas respectively, were fully humidified (100% humidification, hydrogen and air both were set at 70°C) before feeding with a flow rate of 120 sccm for H_2_ and 800 sccm for air. The back pressure for both the anode and cathode were 30 psi.

### Characterizations of the MEAs

The morphology of the Ir@Pt/C catalyst, which was washed off from the cathode MEA by ethanol and was observed using a high-resolution transmission electron microscope (JEOL JEM-2010HR, Japan) operated at 300 kV. High-angle annular dark field (HAADF) images and energy dispersive spectrometer (EDS) elemental line scan analysis were obtained using scanning transmission electron microscopy (STEM) mode on an aberration-corrected FEI Titan G^2^ 60–300 field emission transmission electron microscope (FEI), operated at 300 kV (a max~100 mrad). The nanoparticle crystal structure was determined by X-ray diffraction (TD-3500, Tongda, China) using filtered Cu Kα radiation at 40 kV and 30 mA. The 2θ region between 20° and 80° was measured at a scan rate of 4° min^−1^. X-ray photoelectron spectroscopy (XPS) on a PerkinElmer PHI1600 system (PerkinElmer, USA) using a single Mg Kα X-ray source operating at 300 W and 15 kV. The XPS spectra (BEs) were calibrated using the C 1 s peak of graphite at 284.6 eV as the reference.

## Results and discussion

**Figure 1:**
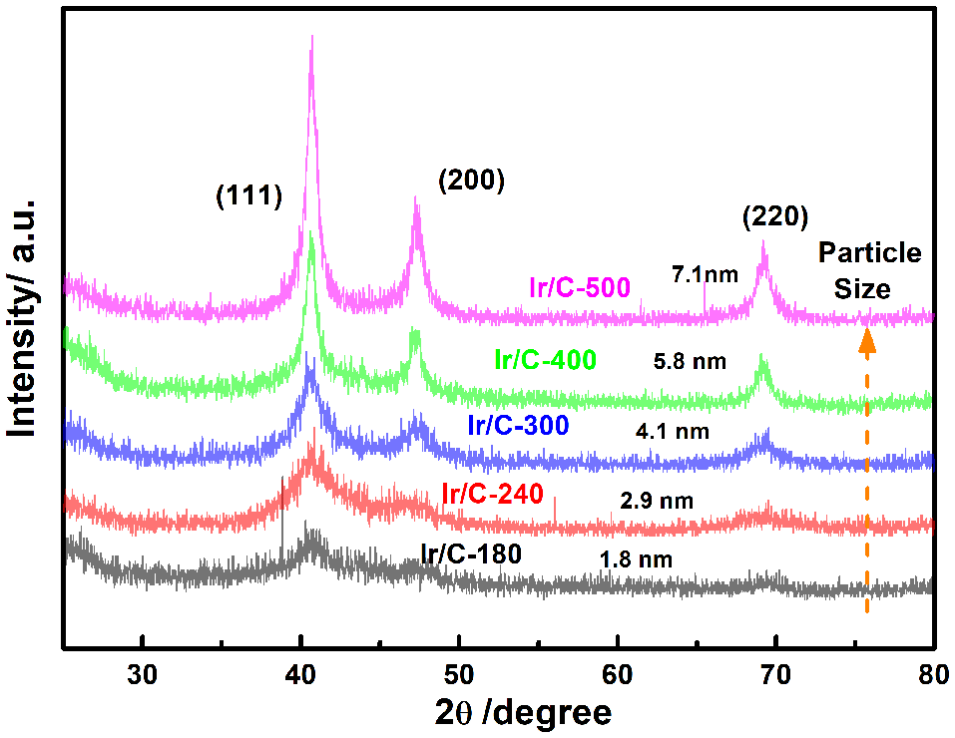
X-ray diffraction patterns of Ir/C with different temperature.

**Figure 2:**
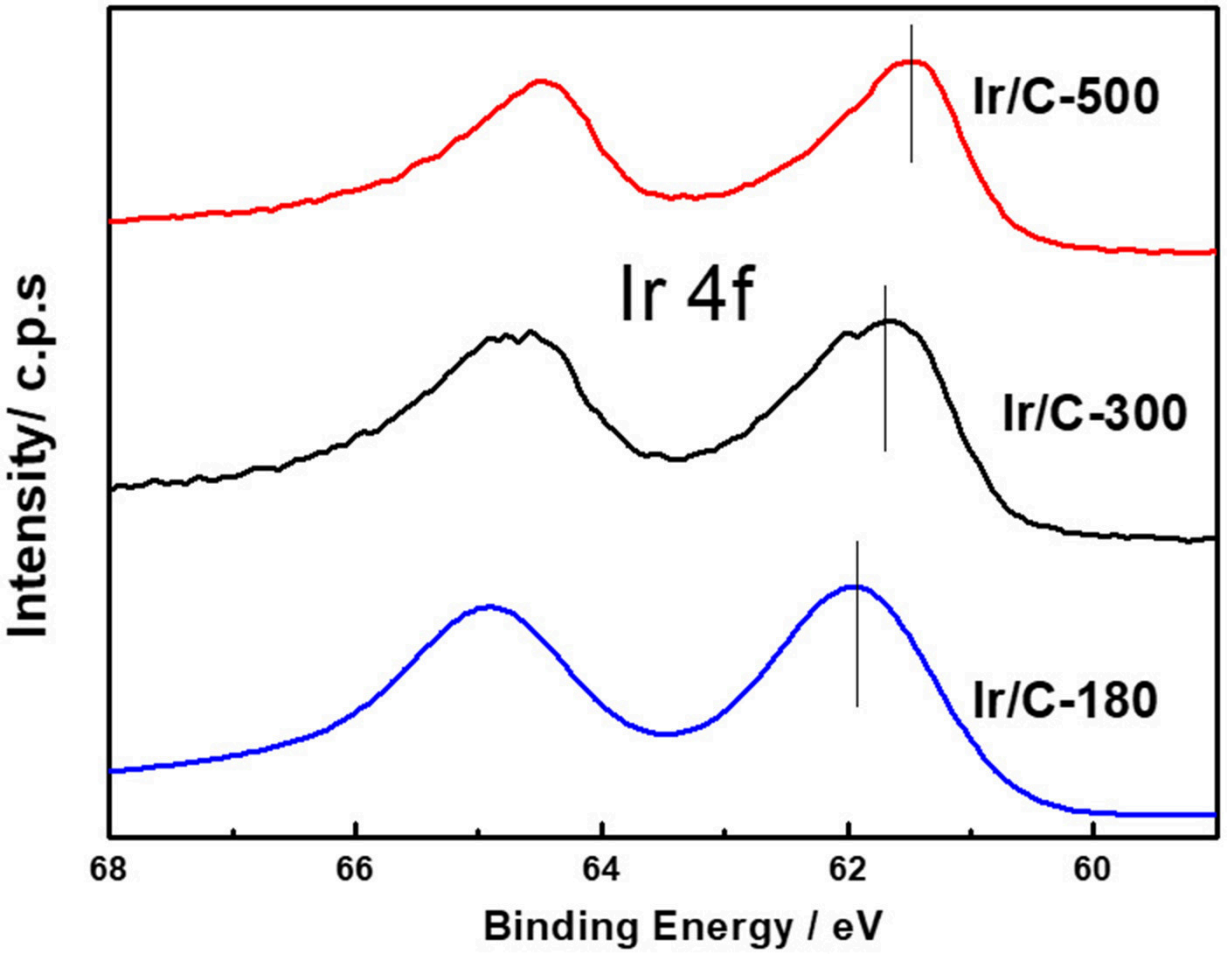
Representative XPS spectra for the Ir 4f core level of Ir/C catalysts.

Figure 1 shows the XRD patterns of Ir/C catalysts annealed at various temperatures. For all the samples, the diffraction peaks at 2θ of 40.6°, 47.31°, and 69.1° can be indexed, respectively, to the (111), (200), and (220) planes of face-centered cubic (fcc) iridium. The expected sharper and more intense diffraction peaks observed as the temperature rose from 180 to 500°C were caused by an increase in the extent of crystallization and particle agglomeration at higher temperatures. The particle sizes, which were calculated from the XRD data by Jade software, were 1.8, 2.9, 4.1, 5.8, and 7.1 nm for Ir/C-180, Ir/C-240, Ir/C-300, Ir/C-400, and Ir/C-500, respectively. The (111) diffraction peak was used to calculate the lattice parameters, which are listed in Table 1. It can be seen that the parameters were almost the same for each sample, indicating that the lattice was not affected by the annealing temperature when it increased from 180 to 500°C. However, the 4f binding energy of Ir in the samples clearly shifted with the annealing temperature or particle size (see Figure 2 and Table 1). As the annealing temperature/particle size increased, the binding energy of Ir 4f decreased, which is quite consistent with previous reports from other groups (Kuznetsova et al., 2012).

**Table 1:** Crystallite sizes and lattice parameters from XRD measurements and binding energies from XPS measurements of Ir/C series catalysts.

**Catalyst**	**Crystallite size (nm)**	**Lattice parameter (Å)**	**Binding energy (eV)**
Ir/C-180	1.8	3.8450	61.91
Ir/C-240	2.9	3.8452	N/A
Ir/C-300	4.1	3.8453	61.68
Ir/C-400	5.8	3.8451	N/A
Ir/C-500	7.1	3.8449	61.49

**Figure 3:**
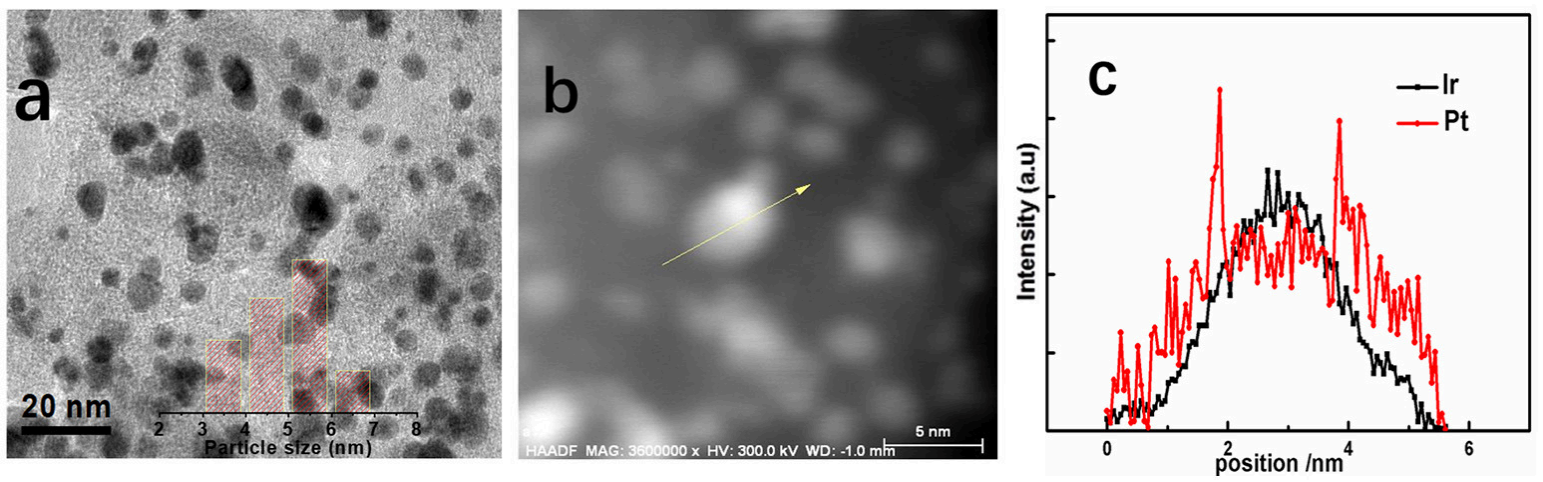
TEM images and the corresponding particle size histograms (inset) of Ir@Pt/C-300 **(a)**; HAADF STEM image of a single Ir@Pt/C-300 particle **(b)** and the corresponding EDS line scan profiles **(c)**.

Figure 3 presents the TEM images of Ir@Pt/C-300 catalysts to get information about structure and morphology changes after the Pt deposition. Typically, the catalysts for the TEM analysis were peeled off from the MEA's surface by ethanol. The Ir@Pt-300 nanoparticles shown in Figure 3a were homogenous dispersed on the carbon support with a narrow size distribution. The average particle size of Ir@Pt-300 nanoparticles was calculated to be 5.4 nm.

In order to further confirm the core-shell structure of Ir@Pt/C-300 catalysts, the high-angle annular dark-field (HAADF) TEM and EDS line scan on a single nanoparticle were performed as shown in Figures 3b,c. It can be seen that Pt signal was highly populated at the edge of particle while Ir signal was concentrated at the center, which clearly manifested the core-shell structure of our Ir@Pt/C-300 catalyst.

**Figure 4:**
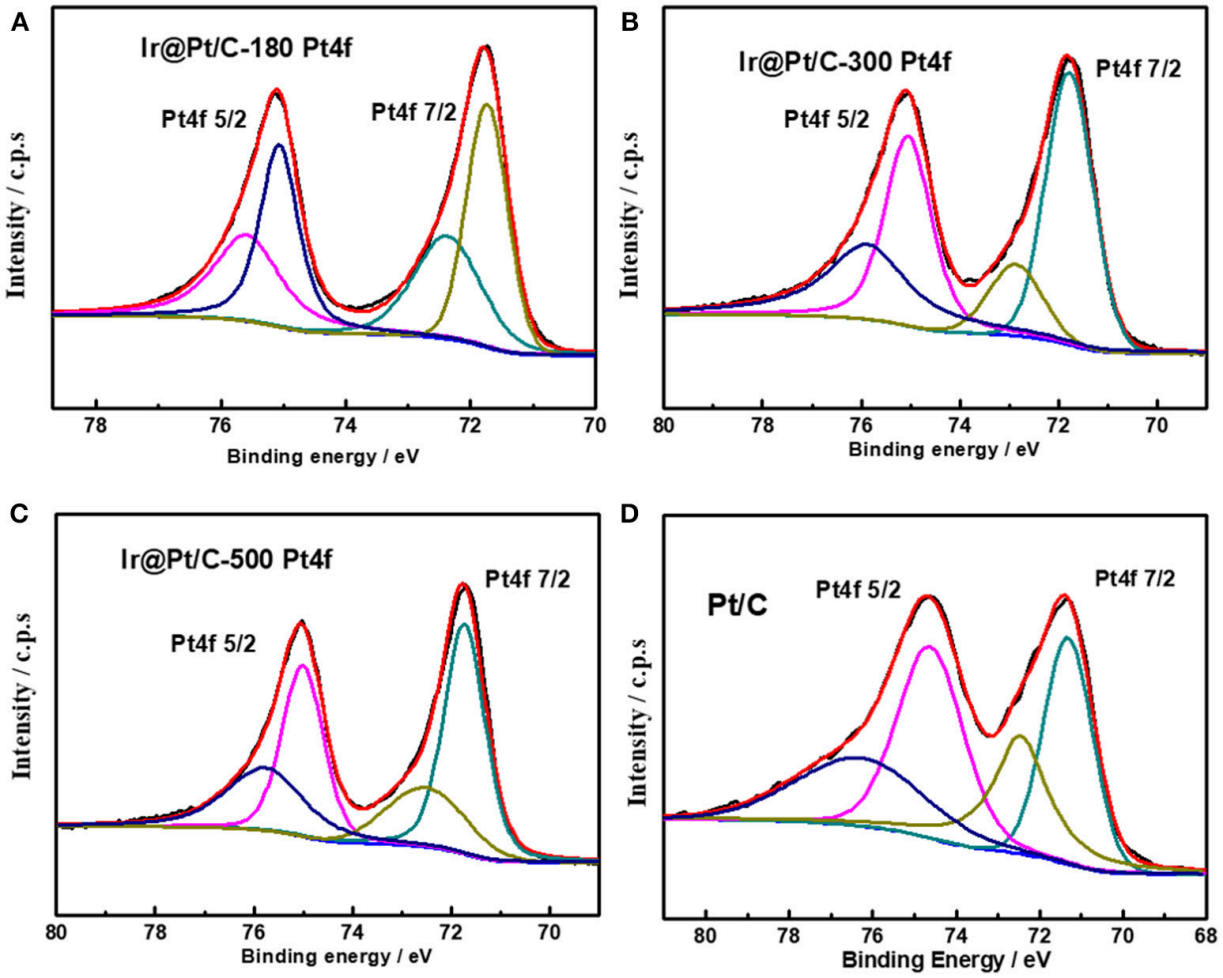
XPS spectra of Pt 4f in **(A)** Ir@Pt/C-180, **(B)** Ir@Pt/C-300, **(C)** Ir@Pt/C-500, and **(D)** Pt/C.

XPS results revealed that the particle size of the Ir core nanoparticles significantly affected the electronic structure of the catalysts. After Pt deposition on the different Ir cores, the core-level binding energies of Pt 4f differed from those in monometallic Pt, as shown in Figure 4 and summarized in Table 2. All the Pt 4f signals of the Ir@Pt/C samples exhibited a positive shift compared to the Pt/C catalysts. However, the Pt 4f binding energy of Ir@Pt/C-300 (71.82 eV) was higher than that of Ir@Pt/C-180 (71.73 eV) and Ir@Pt/C-500 (71.76 eV), suggesting a considerable tuning of the Pt surface electronic structure of Ir@Pt/C-300. In addition, as Figure 4 shows, the Ir@Pt/C-300 catalyst exhibited a higher proportion of Pt^0^ (76.7%) than the rest of the catalysts: Ir@Pt/C-180 (56.7%), Ir@Pt/C-500 (69.5%), and commercial Pt/C (54.5%). It has been reported that the Pt^0^ fraction is a crucial factor associated with ORR catalysis; the relatively high proportion of metallic Pt in Ir@Pt/C-300 implies that it had weaker oxophilicity than the other catalysts, resulting in high ORR performance (Dang et al., 2015). On the other hand, the Ir 4f binding energy of the Ir@Pt/C series MEAs tells a different story. After Pt deposition, the Ir 4f 7/2 values for Ir@Pt/C-180 and Ir@Pt/C-300 shifted negatively compared to Ir/C-180 and Ir/C-300, but the value for Ir@Pt/C-500 shifted positively, indicating an interaction between the Pt shell and the Ir core, and that the interaction varied with the particle size of the Ir core NPs. The slight decrease in Ir 4f binding energy for Ir@Pt/C-300 suggests electron transfer occurred between Pt and Ir, which may have changed the d-band centers of the surface metals and thus improved their surface adsorption and desorption behavior in the ORR process.

**Table 2:** Binding energies (BE) of Pt 4f 7/2 and Ir 4f 7/2 for Pt/C, Ir@Pt/C-180, Ir@Pt/C-300, and Ir@Pt/C-500, with the fraction of Pt^0^ and Ir^0^ content in each catalyst.

**Catalyst**	**BE of Pt^0^4f7/2 /eV**	**Pt^0^/Pt^2+^**	**BE of Ir^0^4f7/2 /eV**	**Ir^0^/Ir^4+^**
Pt/C	71.32	54.5/45.5	/	/
Ir@Pt/C-180	71.73	56.7/43.3	61.18	63.4/36.6
Ir@Pt/C-300	71.82	76.7/23.3	61.39	65.7/34.3
Ir@Pt/C-500	71.76	69.5/30.5	61.59	63.7/36.3

In order to investigate the surface structure and to explore the electrochemical surface area (ECSA) of different catalysts, cyclic voltammograms was implemented in the cell system. As shown in Figure S1, CV plots of different cathode MEAs were measured in a single cell. It can be observed that JM Pt/C MEA prepared by CCM method, has the largest ECSA (86.1 m^2^ g^−1^) since there may be too much contact between Pt atoms and Nafion® in the catalyst layer. As for Ir@Pt/C MEA realized by pulse electrochemical deposition approach, the ECSAs were smaller than that of JM Pt/C MEA. It should be mentioned that every Pt atom would be reduced on the surface of Ir nanoparticle at triple-phase area and establish an appropriate contact to the nearby Nafion® ionomer rather than fully wrapped in it. Besides, it can be observed that when the Ir core size increases from 1.8 to 7.1 nm, the intensity of the H_2_ desorption peak decreases and thus the calculated ESCAs of this series Ir@Pt/C MEA became smaller with the same Pt amounts. Generally, increasing the Ir core size would lead to the decrease of the surface area of Ir nanoparticle, and therefore it could lower the ECSA of Ir@Pt/C catalysts. Furthermore, the positive shift of Pt reduction peak of the Ir@Pt/C MEA at around 0.8 V was also discovered. This may be a sign of Pt coverage on the Ir core and implies that Pt oxides are less likely to form on the Ir core and thus tune the catalytic activity of the core-shell structured catalysts.

**Figure 5:**
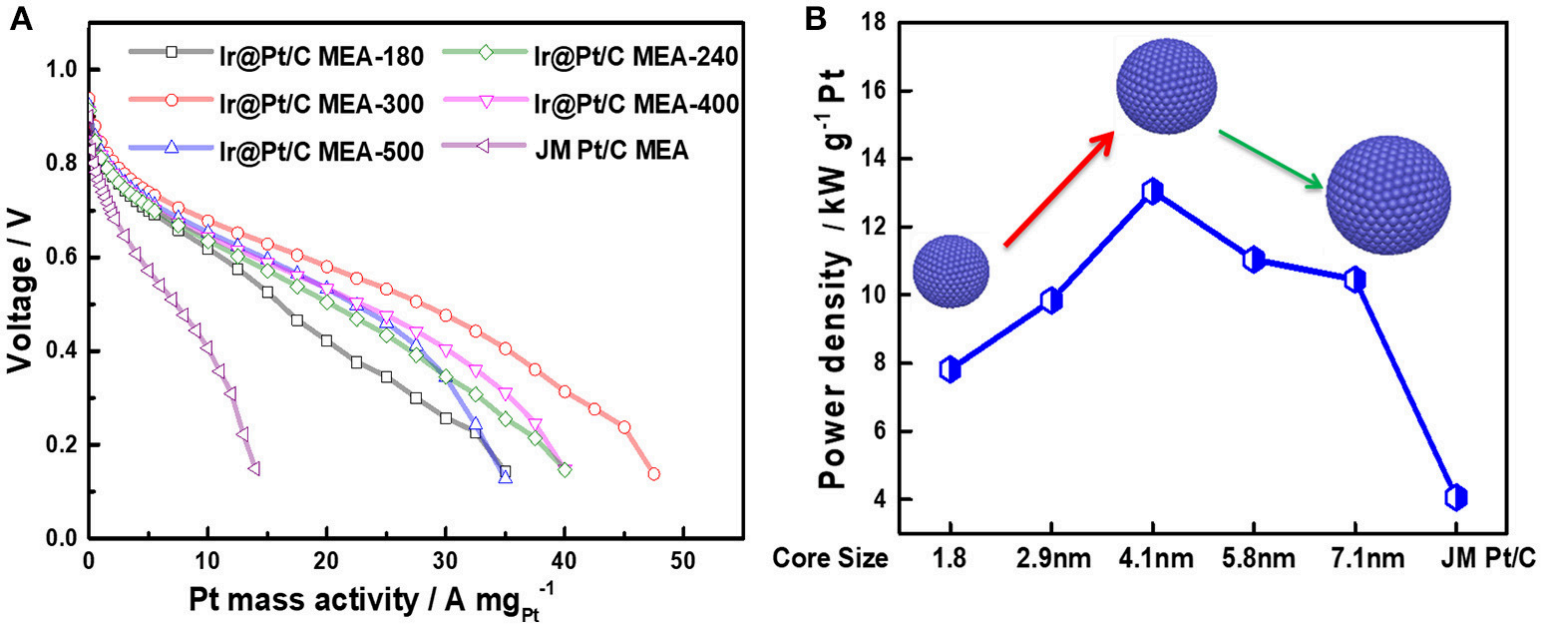
Polarization curves of MEAs **(A)** and the maximum power densities with the change in Ir core sizes **(B)**. All the MEAs have the same anode Pt loading (JM Pt/C 0.1 mg_Pt_ cm^−2^). For Ir@Pt/C MEA cathode: 0.04 mg_Pt_ cm^−2^ and 0.039 mg_Ir_ cm^−2^; for JM Pt/C MEA cathode: 0.1 mg_Pt_ cm^−2^.

Through decorating of platinum atoms on the Ir nanoparticles, a series of Ir@Pt/C MEA were successfully prepared by PED method with ultra-low Pt loadings down to 0.04 mg_Pt_ cm^−2^ at cathode. The anode with Pt loading of 0.1 mg_Pt_ cm^−2^ was prepared using JM 40% Pt/C catalysts. Figure 5A shows the polarization curves obtained by Pt mass with different Ir@Pt/C MEAs and JM Pt/C MEA. As shown in the Figure 5A, the Ir@Pt/C-300 MEA exhibited a best cell performance and Pt utilization efficiency in the whole polarization region whereas JM Pt/C MEA resulted in the poorest performance. With the increasing of Ir core size (from 1.8 to 4.1 nm), the cell performances have been substantially improved. It is interesting that the cell performance then slightly deceased when further elevating the heating temperature from 300 to 500°.

In Figure 5B, the maximum power density of Ir@Pt/C MEAs and JM Pt/C MEA were calculated and benchmarked by Pt loading. It reveals that there is a solid improvement in Pt utilization with Ir@Pt/C-300 at the cathode compared to that of the JM Pt/C MEA cathode. The Figure 5B also exhibited the correlation between maximum power densities and Ir core size. The maxmum mass activity increases and reaches its peak value at Ir core size of 4.1 nm, and then drops slightly when the Ir core size further increases. This indicates that different Ir core sizes leads to distinct cathode catalytic behavior of Ir@Pt/C catalysts, which significantly affect the ORR activity through tuning the surface electronic structure between Ir and Pt.

Figure S2 showed a preliminary durability test of fuel cell with Ir@Pt/C-300 MEA for 100 h under constant discharge process at 800 mA cm^−2^. There was no obvious fluctuation of operation voltage during the test, which revealed good initial stability of our Ir@Pt/C-300 MEA.

## Conclusion

A series of Ir@Pt/C MEAs were successfully prepared through construction of core-shell structured catalysts in the cathode catalyst layer with a facile pulse electrodeposition synthesis route. By optimizing the Ir core size dependent on the thermal treatment, the cell performance of different MEA were investigated and balanced to the acceptable choice. The as-prepared Ir@Pt/C series MEA that contains the ultra-low Pt loading (0.04 mg cm^−2^ at cathode) showed the competitive single cell performance and Pt utilization efficiency compared with those of JM Pt/C MEA (0.1 mg cm^−2^ at cathode). It is suggested that the optimum Ir core size with 4.1 nm shows appropriate crystallinity which will tune the surface electronic structure between Ir and Pt and finally lead to the high cell performance.

## Author contributions

DD and QL conceived the project. H-BZ carried out the experiment and collected the data. LA performed the electrochemical tests. YZ, CQ, and YF made contribution to the analysis and discussion of the results. DD drafted the manuscript, and all authors contributed to the final version of the manuscript.

### Conflict of interest statement

The authors declare that the research was conducted in the absence of any commercial or financial relationships that could be construed as a potential conflict of interest.
