# Supplementary material for: Tuning the Catalytic Activity of Ir@Pt Nanoparticles Through Controlling Ir Core Size on Cathode Performance for PEM Fuel Cell Application
Source: Front Chem. 2018 Jul 26;6:299. doi: 10.3389/fchem.2018.00299 (PMC6070630; doi:10.3389/fchem.2018.00299)
Supplement: Supplementary file 1 [file Data_Sheet_1.pdf]

Tuning the catalytic activity of Ir@Pt nanoparticles through controlling Ir core size on cathode performance for PEM fuel cell application

Hao-Bo Zheng [a], *Lu An* [a], *Yuying Zheng* [a], *Chong Qu* [b], *Yanxiong Fang* [a], *Quanbing Liu* [a*], *Dai Dang* [a*]

[a] School of Chemical Engineering and Light Industry, Guangdong University of Technology, Guangzhou, Guangdong, 510006, China

[b] Department of Materials Science and Engineering College of Engineering, Peking University, Beijing 100871, China

*Corresponding author:

Quanbing Liu, School of Chemical Engineering and Light Industry, Guangdong University of Technology, Guangzhou 510006, China

Dai Dang, School of Chemical Engineering and Light Industry, Guangdong University of Technology, Guangzhou 510006, China

Tel: +86-20-39322812

E-mail: liuqb@gdut.edu.cn; dangdai@gdut.edu.cn

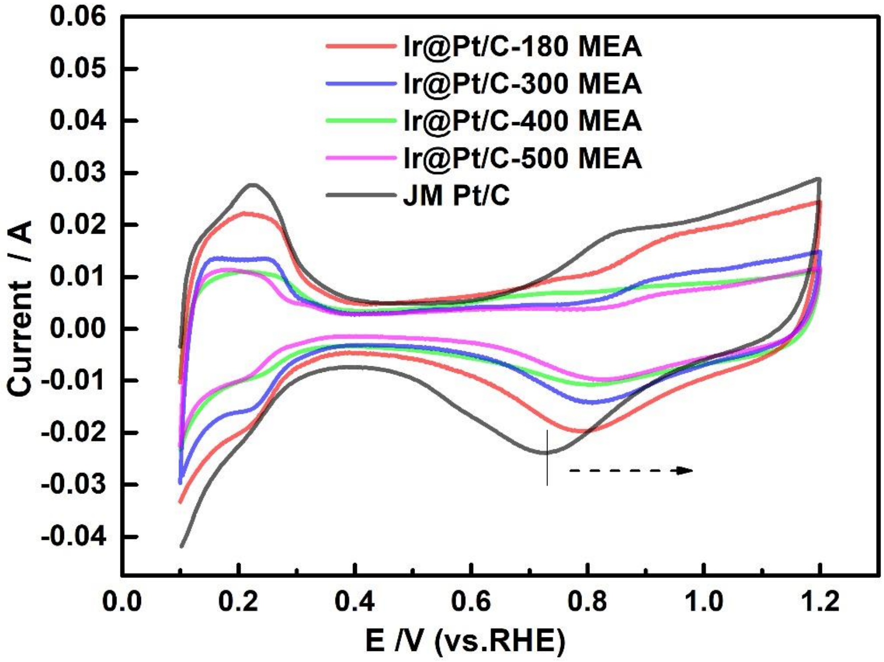

Figure. S1 Cyclic voltammograms of the MEAs with different cathodes. The experiments were conducted in a single cell at room temperature, using humidified N_2 at the cathode (working electrode) and humidified H_2 at the anode (counter electrode cum reference electrode). Scan rate: 50 mV s^{-1}

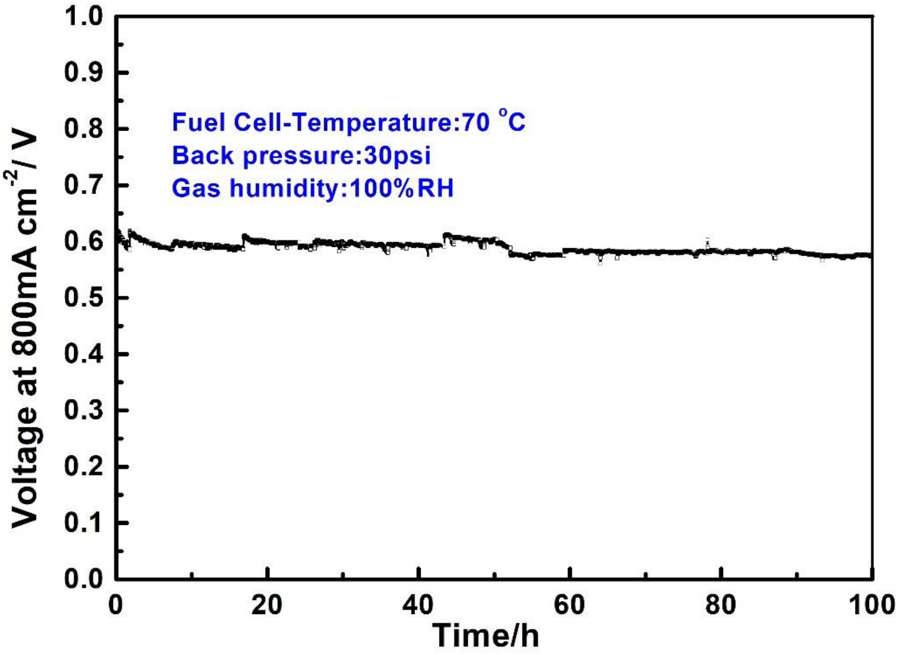

Figure. S2 Preliminary stability test of the Ir@Pt/C-300 MEA at 800 mA cm^{-2} for 100 h
